# Soybean NADP-Malic Enzyme Functions in Malate and Citrate Metabolism and Contributes to Their Efflux under Al Stress

**DOI:** 10.3389/fpls.2017.02246

**Published:** 2018-01-10

**Authors:** Ying Zhou, Zhenming Yang, Yuezi Xu, Haoran Sun, Zhitao Sun, Bao Lin, Wenjing Sun, Jiangfeng You

**Affiliations:** Laboratory of Soil and Plant Molecular Genetics, College of Plant Science, Jilin University, Changchun, China

**Keywords:** aluminum toxicity, anaplerotic reaction, citrate efflux, malic enzyme, tricarboxylic acid cycle

## Abstract

Malate accumulation has been suggested to balance Al-induced citrate synthesis and efflux in soybean roots. To test this hypothesis, characteristics of Al-induced accumulation and efflux of citrate and malate were compared between two soybean genotypes combining a functional analysis of *GmME1* putatively encode a cytosolic NADP-malic enzyme. Similar amounts of citrate were released, and root elongation was equally inhibited before 8 h of Al treatment of Jiyu 70 and Jiyu 62 cultivars. Jiyu 70 began to secrete more citrate and exhibited higher Al resistance than did Jiyu 62 at 12 h. A sustained increase in internal malate and citrate concentrations was observed in Jiyu 70 at 24 h of Al treatment. However, Jiyu 62 decreased its malate concentration at 12 h and its citrate concentration at 24 h of Al treatment. GmME1 localized to the cytoplast and clustered closely with cytosolic malic enzymes AtME2 and SgME1 and was constitutively expressed in the roots. Al treatment induced higher NADP-malic enzyme activities and *GmME1* expression levels in Jiyu 70 than in Jiyu 62 within 24 h. Compared with wild-type hairy roots, over-expressing *GmME1* in hairy roots (*GmME1*-OE) produced higher expression levels of *GmME1* but did not change the expression patterns of either of the putative citrate transporter genes *GmAACT1* and *GmFRDL* or the malate transporter gene *GmALMT1*, with or without Al treatment. *GmME1*-OE showed a higher internal concentration and external efflux of both citrate and malate at 4 h of Al stress. Lighter hematoxylin staining and lower Al contents in root apices of *GmME1*-OE hairy roots indicated greater Al resistance. Comprehensive experimental results suggest that sustaining Al-induced citrate efflux depends on the malate pool in soybean root apices. *GmME1* encodes a cytosolic malic enzyme that contributes to increased internal malate and citrate concentrations and their external efflux to confer higher Al resistance.

## Introduction

Aluminum (Al) toxicity seriously restricts crop yield in acidic soils, which cover almost 40% of the arable land worldwide (Ma et al., 2001). Al can significantly inhibit root growth and disrupt root function rapidly (Delhaize and Ryan, 1995). Fortunately, some plant species have developed Al resistance mechanisms to grow in acidic soils. Al-induced organic acid efflux has been well established to detoxify Al internally and externally and thus far is the best-documented Al resistance mechanism in higher plants (Ma et al., 2001; Ryan et al., 2001; Kochian et al., 2004; Kochian et al., 2015). Two patterns have been classified according to the rapidity of organic acid release (Ma et al., 2001). In pattern I, some plant species, such as wheat (Ryan et al., 1995) and buckwheat (Ma and Miyasaka, 1998), can rapidly release malate or oxalate, respectively, after Al stress. In pattern II, some species, such as *Cassia tora* (Ma et al., 1997) and soybean (Yang et al., 2001), secrete citrate after at least 4 h of Al treatment. In both patterns, organic acid anion transporters are crucial for organic acid efflux under Al stress and for Al resistance (Ryan et al., 2011). Over-expression of *TaALMT1* (Aluminum-activated malate transporter) in barley (Liu et al., 2009) and wheat (Collins et al., 2008) increased Al resistance by 8-fold and 20-fold, respectively. The Al resistance of Arabidopsis can be increased by 2.5-fold and 3-fold by over-expression of MATE family citrate transporter genes *SbMATE* (Magalhaes et al., 2007) and *ZmMATE1* (Maron et al., 2013), respectively.

The strategies to over-express enzymes involved in organic acid metabolism have also been proven effective in regulating Al resistance in some plant species. Over-expression of citrate synthase genes in different plant species, including alfalfa, Arabidopsis, canola and tobacco, can increase their citrate efflux and Al^3+^ resistance in transgenic plants (Koyama et al., 1999; Anoop et al., 2003; Barone et al., 2008; Deng et al., 2009; Han et al., 2009). Malate dehydrogenase genes of different origins were over-expressed in alfalfa (Tesfaye et al., 2001) and tobacco (Wang et al., 2010) and showed enhanced malate efflux and improved Al^3+^ resistance. *SgME1* encoding NADP-dependent malic enzyme was found to functionally control malate synthesis and secretion and thus Al detoxification (Sun et al., 2014). Recently, over-expression of *VuFDH* encoding a mitochondrial formate dehydrogenase and *VuAAE3* encoding Acyl activating enzyme 3 in tobacco were found to increase Al tolerance by decreasing formate production and oxalate accumulation, respectively (Lou et al., 2016a,b).

Our previous study showed that Al-induced citrate secretion from soybean required almost 4 h of Al exposure (Yang et al., 2000, 2001), which was clearly classified as pattern II (Ma, 2000). Soybean mitochondrial enzymes, including increased citrate synthase and decreased aconitase, were found to contribute to the citrate efflux from roots under Al stress (Xu et al., 2010). Sustained Al-induced citrate efflux from common bean, the close relative of soybean, was reported to rely on the maintenance of high citrate synthase activity and citrate pool (Rangel et al., 2010). Cytosol phosphoenolpyruvate carboxylase (PEPC) and mitochondrial NAD malic enzyme were suggested to contribute to the accumulation and the secretion of citrate in common bean by fueling the tricarboxylic acid (TCA) cycle (Rangel et al., 2010).

Organic acid metabolism-related enzymes was proposed to contribute to detoxifying Al in some plant species (Rangel et al., 2010; Xu et al., 2010; Sun et al., 2014; Lou et al., 2016a,b). In soybean, during the process of citrate efflux from soybean, malate but not citrate significantly decreased with the increase in Al treatment duration (Yang et al., 2001). Thus, malate was hypothesized to maintain balance between the citrate pool and efflux in the soybean roots exposed to Al. However, there is no direct evidence to support this hypothesis until now. Malate is tightly controlled to affect a series of physiological processes because it is at the branching point of many metabolic pathways (Santelia and Lawson, 2016). The transcript level of NADP-malic enzyme was found by microarray assay to increase in soybean root apices under Al stress (You et al., 2011). In this study, in order to elucidate the role of malate pool in the Al-induced citrate efflux from soybean, *GmME1*, probably encoding NADP-dependent malic enzyme in soybean, was functionally characterized to evaluate its possible implications in organic acid pool and efflux. Al-induced accumulation and efflux of citrate and malate were also compared between two soybean genotypes in relation to GmME1 enzyme activities and gene expression patterns.

## Materials and Methods

### Hydroponic Culture and Al Treatment Conditions

Our previous work has shown that soybean cultivar Jiyu 70 and Jiyu 62 exhibited contrast Al resistance capabilities, thus was used as Al tolerant and Al sensitive cultivars respectively in our lab. Seeds of soybean Jiyu 70 and Jiyu 62 cultivars were germinated in darkness for 3 days. Then, seedlings with roots 4–5 cm long were selected for transplant into 0.5 mM CaCl_2_ solution. After 24 h of culture, seedlings were exposed to 0.5 mM CaCl_2_ solution containing 0 or 30 μM AlCl_3_ (pH 4.5). Root length was measured at 0, 8, 12, and 24 h. The relative root elongation (RRE) was calculated to evaluate Al sensitivity. The formula is root elongation with AlCl_3_ treatment/root elongation, without AlCl_3_ × 100.

The remaining germinated seedlings were grown in 1-L plastic pots filled with nutrient solution with composition, as described by Horst et al. (1992). The solutions were modified to pH 4.5 by HCl and aerated continually. After 14 days of culture, seedlings were pre-cultured in 0.5 mM CaCl_2_ solution (pH 4.5) overnight and then transferred to 0.5 mM CaCl_2_ solution containing 0 or 30 μM AlCl_3_ (pH 4.5). Treatment solutions were refreshed at 2, 4, 8, 12, and 24 h and collected, respectively, for organic acid analysis. Root exudates were concentrated and purified, as described in Ma et al. (1997). Simultaneously, root apices were excised from the parallel Al-treated soybean seedlings at 0, 2, 4, 8, 12, and 24 h (∼0.5 g for each sample). Citrate and malate were extracted from the excised root apices, as described in Yang et al. (2001). Their concentrations were measured by high-performance liquid chromatography (HPLC) (LC 20AT, Shimadzu, Tokyo, Japan) with a Shodex RSpakKC-811 ion-exclusion column (300 × 8 mm, Shimadzu, Tokyo, Japan). NADP-malic enzyme (EC1.1.1.40) was extracted and quantified by an NADP-ME kit (Comin Biotechnology, Suzhou, China). The rate of increase of NADP was monitored at 340 nm.

The 7-day-old seedlings were transferred into 0.5 mM CaCl_2_ solution (pH 4.5) overnight and then exposed to 0.5 mM CaCl_2_ solution (pH 4.5) including 0 or 30 μM AlCl_3_. Then, 0- to 1-cm root apices were excised at a treatment duration of 0, 2, 4, 8, 12, and 24 h. The collected root apices were immediately placed in liquid nitrogen and stored at -80°C for RNA isolation.

Soybean seedlings were cultivated in a controlled environment with a 14 h/25°C day and 10 h/22°C night cycle. Light intensity was controlled as 300 μmol m^-2^s^-1^. Relative humidity was kept at 60%.

Jiyu 70 was sown in the field of the agricultural trial station of Jilin University at the end of April 2014. The soil contained 49.4 ± 4.8 g/kg available nitrogen, 11.8 ± 4.1 g/kg available P, 170 ± 6.2 g/kg K, and 21.8 ± 3.7 g/kg organic carbon at pH 6.5. After 18 days, the roots, shoots, leaves, flowers and pods were sampled in the field-grown soybean. The samples were stored at -80°C for RNA isolation.

### Gene Transcriptional Expression

RNA was extracted from root apices by Trizol reagent (Invitrogen, Carlsbad, CA, United States). cDNA was obtained by reverse transcribing with M-MLV reverse transcriptase (TaKaRa Bio, Tokyo, Japan). The gene-specific primers were designed according to the CDS of *GmME1* (Glyma.06G087800) by Primer 3.0 online^1^ and had the following sequences: forward primer 5′-AGCATCTGTGGTATTAGCA-3′; reverse primer 5′-GGAATAAGAAGGTATGGTCAAC-3′. The housekeeping gene β-*Tublin* (GenBank ID: 100811275) had the following primer: forward primer 5′-GGAAGGCTTTCTTGCATTGGTA-3′; reverse primer 5′-AGTGGCATCCTGGTACTGC-3′. Quantitative real-time PCR (qRT-PCR) was conducted in an Mx3005P machine (PRIMER Biosoft Company, Palo Alto, CA, United States). The 25 μl reaction system included 2 μl of cDNA template (50 ng), 1 μl of a mixture of forward and reverse primers (10 mM), 12.5 μl of 2× SYBR Taq (TaKaRa, Bio Inc.), and 9.5 μl of milli-Q water. The program was as follows: 95°C for 30 s; 30 cycles of 95°C for 5 s, 60°C for 20 s, 95°C for 60 s, 55°C for 30 s, and 95°C for 30 s. Relative expression was computed according to the 2^-ΔΔC_t_^ method (Livak and Schmittgen, 2001).

### Gene Cloning and Sequence Analysis

PCR was performed with cDNA template prepared by root apices treated with Al for 4 h. The primers were designed according to the CDS of *GmME1* (Glyma.06G087800), considering the vector pCAMBIA3301 with BamHI with the following primer sequence: GmME1-NF5′-CATTCTGGCGGGATCCGCAGCAGCAGCAGCAATGTCGAGCGCTTCGTTGA-3, BamHI; GmME1-NR5′-GAGAAAGCTTGGATCCAACGGTAGCTTCGGTAGCCT-3′, BamHI. The PCR products were purified using the TransGen Biotech Kit according to the manufacturer’s protocol, confirmed by sequencing, and aligned to vectors (pCAMBIA3301) by in-fusion enzyme. Phylogenetic tree construction and sequence comparison were conducted with MEGA 5.1 and Cluster. Other MATE family gene sequences were blasted at the NCBI website as follows: *Arabidopsis thaliana* (*AtNADP-ME1* GeneID:816509, *AtNADP-ME2* GeneID:831039, *AtNADP-ME3* GeneID:832657, *AtNADP-ME4* GeneID:844314), *Flaveria bidentis* (*FbNADP-ME* LOCUS: AAW56450), *Lycopersicon esculentum* (LeME2 LOCUS: AAB58728), *Medicago truncatula* (*MtNADP-ME* GeneID:25490143), *Nicotiana sylvestris* (*NtNADP-ME* GeneID:104247285), *Oryza sativa* (*OsNADP-ME* GeneID:4338007), *Stylosanthes guianensis* (*SgME1* LOCUS AGH32501), and *Vigna Umbellata* (*VuNADP-ME* LOCUS CAA56354).

The subcellular localization of GmME1 was determined as follows: The CDS of *GmME1* was cloned into pENSG-N-YFP vector with the cauliflower mosaic virus (CaMV) 35S as a promoter. The resulting constructs were fully sequenced to check the sequence accuracy. Plasmid DNA was transformed into Arabidopsis protoplast cells. The imaging of GFP fluorescence was conducted by microscopy (Zeiss 2012 Observer, Göttingen, Germany).

### Agrobacterium-Mediated Over-expression of *GmME1* in Soybean Hairy Roots

With CaMV 35S as the promotor, PCR product was cloned into the modified pCamBIA3301 vector. After verification by sequence, the resulting construct was transformed into the K599 strain by electroporation. Soybean transformation in Jiyu 62 cotyledons and hairy root induction were performed according to Subramanian et al. (2005). Hairy roots with scanning luciferase activity greater than 3000 were considered successfully transformed. The hairy roots induced by only K599 were considered wild type (WT). Both transgenic and WT hairy roots were treated in 0.5 mM CaCl_2_ solution (pH 4.5) including 0 or 30 μM AlCl_3_ in a 5-ml plastic tube. Root exudates were collected at 4 h for citrate and malate efflux measurement. Root apices (0–1 cm) were cut, and three were stained by hematoxylin. The remaining root apices were stored at -80°C for RNA isolation, internal organic acid concentration measurement, or Al concentration examination. Internal citrate and malate were extracted according to Yang et al. (2000). Citrate and malate concentrations and efflux were measured by enzymatic method (Delhaize et al., 1993). The Al concentration in hairy root apices was determined by 2 M HCl and assayed by an atomic absorption spectrophotometer equipped with a graphite furnace atomizer (Perkin Elmer AAnalyst 700, United States).

## Results

### Time Course of Relative Root Elongation and Citrate Efflux in Two Soybean Genotypes under Al Stress

**FIGURE 1:**
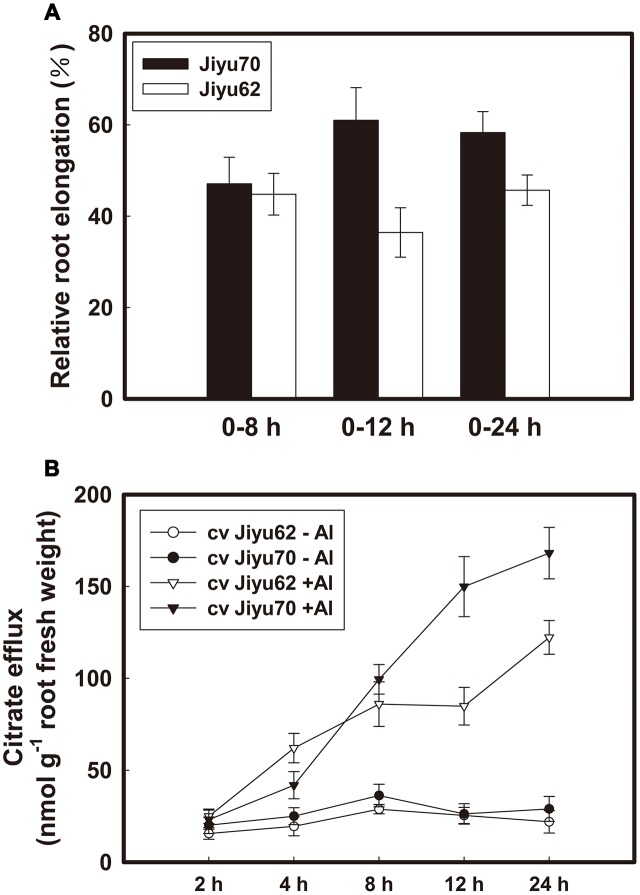
Effect of Al on root elongation **(A)** and Al-induced citrate efflux **(B)** of two soybean genotypes. Seedlings with roots 4–5 cm long were exposed to 0 or 30 μM Al in 0.5 mM CaCl_2_ solution. The root length was measured with a ruler at 0, 8, 12, and 24 h. Fourteen-day-old seedlings were exposed to 0 or 30 μM Al in 0.5 mM CaCl_2_ solution. Root exudates were collected at 2, 4, 8, 12, and 24 h. Relative root elongation was calculated, and organic acid exudates were analyzed as described in Section “Materials and Methods.” The values are the means of three independent experiments ± SDs.

Root elongation was nearly equally inhibited during 8 h of Al exposure for both genotypes, whereas recovery began at 12 h for Jiyu 70 and at 24 h for Jiyu 62 (**Figure 1A**). A significant difference in Al-induced citrate exudation (**Figure 1B**) was found between Jiyu 70 and Jiyu 62 at 12 h of Al treatment. The RRE of Jiyu 70 was approximately 1.7 fold greater than that of Jiyu 62 at 12 h (**Figure 1A**). In addition, an approximately 1.8 fold greater Al-induced citrate efflux was found in Jiyu 70 than in Jiyu 62 (**Figure 1B**). The higher Al resistance of Jiyu 70 depends on the recovery from Al-induced root elongation inhibition, in which maintenance of continuous citrate efflux is necessary. Quimbaya, an Al-resistant common bean genotype, was also found to recover root elongation inhibition by sustaining Al-induced citrate efflux (Rangel et al., 2010).

### Time Course of Internal Citrate and Malate Concentration and NADP-Mali Enzymes Activities in Two Soybean Genotypes under Al Stress

**FIGURE 2:**
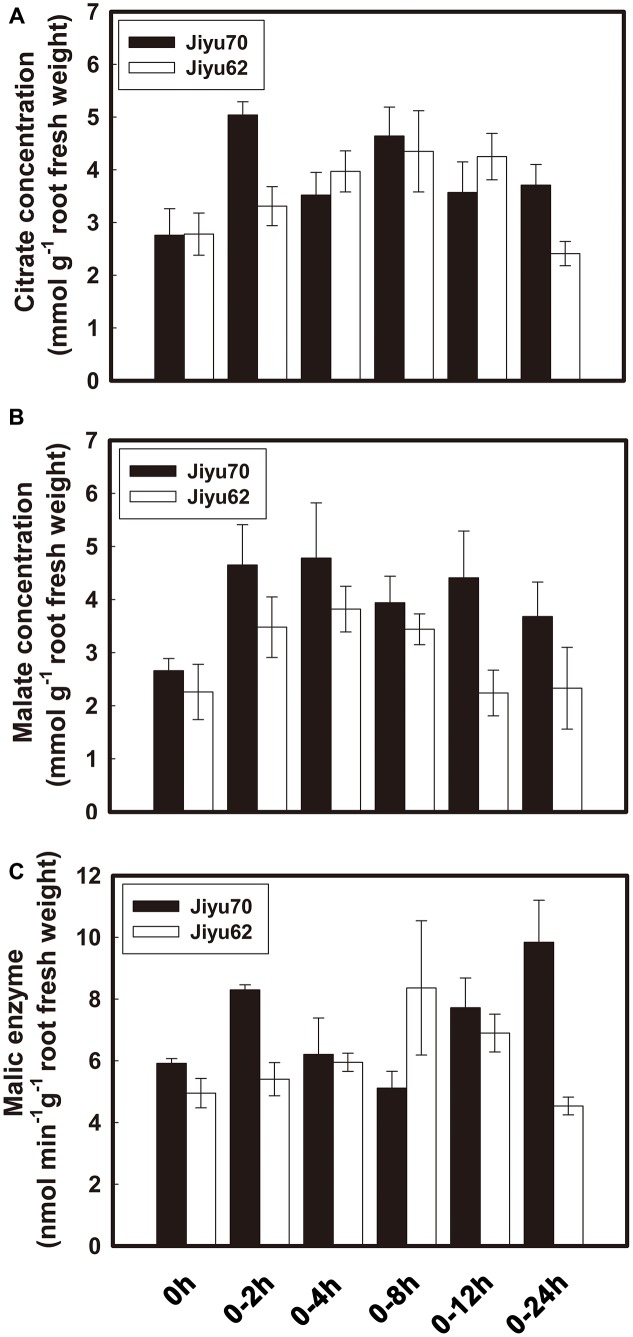
The time course of internal citrate **(A)**, malate **(B)**, and NADP-malic enzyme **(C)** concentration in root apices of two soybean genotypes. Soybean seedlings were transferred to 0.5 mM CaCl_2_ solution (pH 4.5) with or without 30 μM AlCl_3_. The root apices of soybean were excised at 2, 4, 8, 12, and 24 h. Internal malate, citrate and malic enzyme were extracted and examined. Error bars represent ± SD (*n* = 3).

Al treatment increased the internal citrate and malate concentrations beginning at 2 h of Al exposure for both genotypes (**Figures 2A,B**). Higher citrate and malate concentrations were always found in Jiyu 70 during throughout the Al treatment duration. A great decrease in the malate concentration occurred at 12 h of Al treatment, followed by a decrease in the citrate concentration at 24 h in Jiyu 62 (**Figures 2A,B**). The exhaustion of malate (**Figure 2B**) might have a negative effect on the citrate concentration (**Figure 2A**) and efflux (**Figure 1B**) under Al stress. Compared with Jiyu 70, malate exhaustion at Jiyu 62 at 12 h was consistent with the lower citrate efflux at 24 h (**Figure 1B**). This result is consistent with our previous study in another soybean genotype, Shuzunari, in which the malate concentration but not the citrate concentration was found to decrease under Al stress (Yang et al., 2001). Malate might contribute to balance citrate synthesis and efflux. Al treatment increased the activities of NADP-malic enzymes beginning at 2 h of Al exposure for Jiyu 70. Activities of NADP-malic enzymes in Jiyu 62 increased at 4 h, peaked at 8 h and decreased in the remaining Al exposure duration (**Figure 2C**). The lower NADP-malic enzyme activities of Jiyu 62 were consistent with its lower malate concentration in root apices (**Figures 2B,C**).

### The Transcriptional Expression of *GmME1*

**FIGURE 3:**
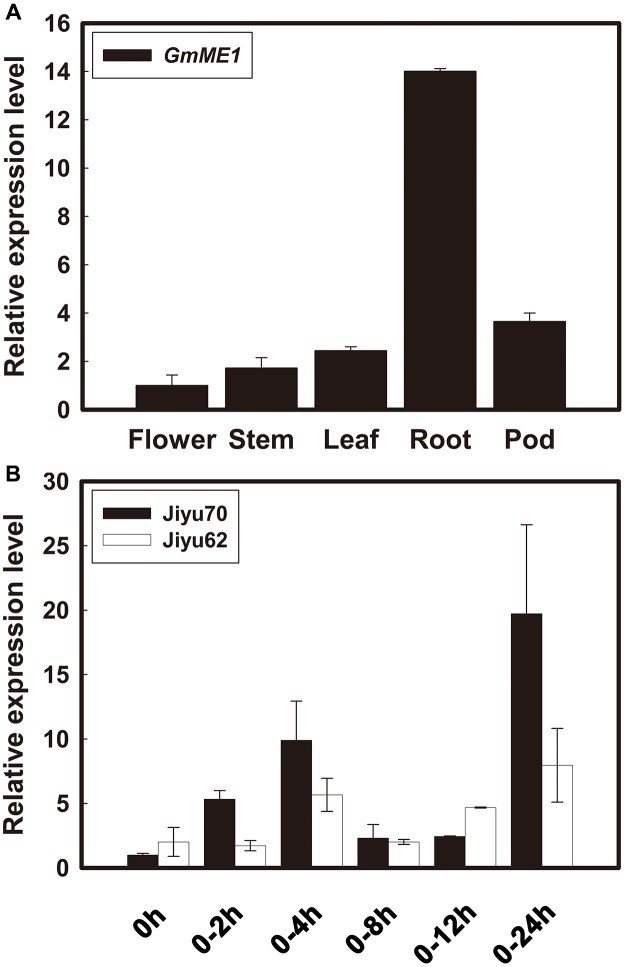
Transcriptional expression patterns of *GmME1* in soybean. **(A)** Tissue localization expression of *GmME1*. After 18 days of growth in the field, the roots, shoots, leaves, flowers and pods were sampled from Jiyu 70 soybean seedlings. **(B)** Four-day-old seedlings were cultured in 0.5 mM CaCl_2_ solution containing 30 μM AlCl_3_ (pH 4.5) for 0, 2, 4, 8, 12, and 24 h. Total RNA was extracted from root apices (0–1 cm). qRT-PCR was performed using the *β*-tubulin gene as an internal standard with 0 h seedling root apices as a calibrator. Data are given as the mean ± SD (*n* = 3).

*GmME1* was constitutively expressed throughout the entire plant of Jiyu 70, especially in the roots (**Figure 3A**). Its transcriptional abundance fluctuated in Jiyu 70 and increased 10 fold and 20 fold at 4 and 24 h, respectively, under Al stress. Jiyu 62 also displayed higher expression at 4 and 24 h, albeit with less magnitude (**Figure 3B**).

### The Bioinformatic Analysis of *GmME1*

Full-length *GmMe1* was isolated from soybean root apices (GenBank: 100778170). *GmMe1* encodes a protein with 619 amino acids. As predicted in http://prosite.expasy.org/scanprosite, GmMe1 contains a malic enzyme signature (Ps00331) at its 331-347 site. Sequence analysis at http://www.cbs.dtu.dk/services/SignalP/ showed that GmMe1 does not contain any predicted organelle sorting signal (data not shown).

**FIGURE 4:**
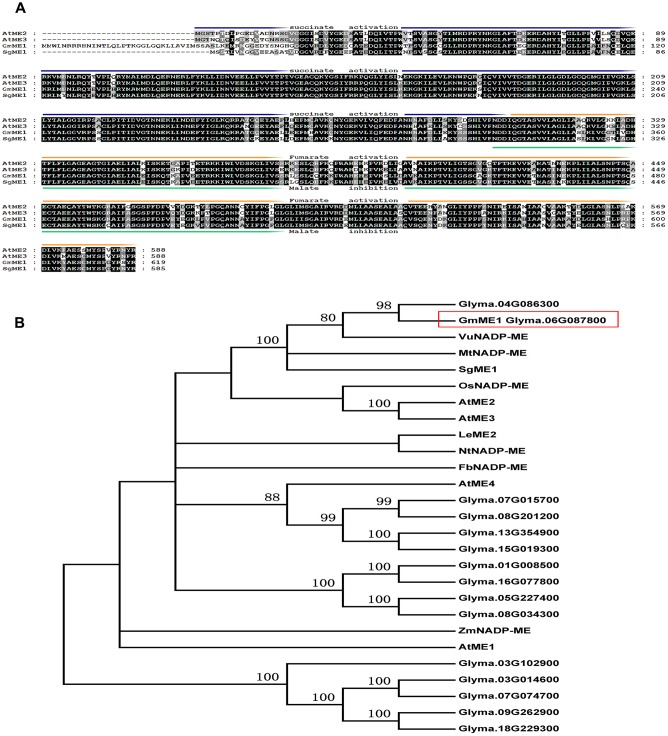
Sequence **(A)** and phylogenetic tree **(B)** analysis of GmME1 and other known plant NADP-Malic enzymes. **(A)** Alignment of the amino acid sequences of GmME1 and orthologous proteins from other plant species, including AtME2 (GeneID:831039), AtME3 (GeneID:832657) and SgME1 (LOCUS AGH32501). Regions of the primary structure of each isoenzyme are labeled as follows: fumarate activation (orange), malate inhibition (green), and succinate activation of the reverse reaction (blue). **(B)** Phylogenetic relationship of GmME1 and other known Malic enzymes proteins. *Arabidopsis thaliana* (*AtNADP-ME1* GeneID:816509, *AtNADP-ME2* GeneID:831039, *AtNADP-ME3* GeneID:832657, *AtNADP-ME4* GeneID:844314), *Flaveria bidentis* (*FbNADP-ME* LOCUS: AAW56450), *Lycopersicon esculentum* (LeME2 LOCUS: AAB58728), *Medicago truncatula* (*MtNADP-ME* GeneID:25490143), *Nicotiana sylvestris* (*NtNADP-ME* GeneID:104247285), *Oryza sativa* (*OsNADP-ME* GeneID:4338007), *Stylosanthes guianensis* (*SgME1*LOCUS AGH32501), *Vigna umbellata* (*VuNADP-ME* LOCUS CAA56354), *Zea mays* (*ZmNADP-ME* GeneID:542209)*1*LOCUS AGH32501) and *GmME1 Glyma.06G087800*).

The cloned *GmME1* showed high similarity to *AtME2* and *AtME3*, with identities of 77 and 76%, respectively. Arabidopsis NADP-malic enzyme isoforms shared high degrees of identity but have very different roles (Wheeler et al., 2008). With minimal structural differences, *AtME2* and *AtME3* display the forward (malate oxidative decarboxylation to decompose malate) and reverse (pyruvate reductive carboxylate ion to produce malate) reactions. GmME1 is conserved at the suggested critical regulatory regions of fumarate activation and malate inhibition (**Figure 4A**). Multiple isoforms of GmME1 might function redundantly or display different roles. GmNADP-ME homologues had identities between 35 and 96% (**Figure 4A**). Phylogenetic analysis showed that GmME1 exhibited high similarity to SgME1 (**Figure 4B**), which suggests contribution to more malate synthesis and efflux under Al stress (Sun et al., 2014). GmME1 also closely clustered with VuNADP-ME and OsNADP-ME (Chen et al., 2015).

### Subcellular Localization of GmME1

**FIGURE 5:**
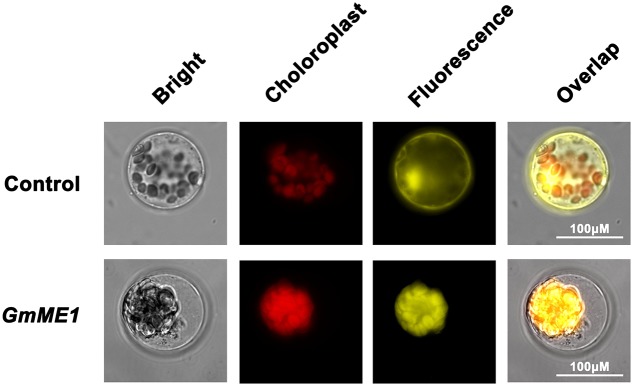
Subcellular localization of *GmNADP-ME.* The YFP alone (above columns) and fusion protein GmME1:YFP (down columns) were transiently expressed in Arabidopsis protoplasts. The panels show the overlapped images of bright-field, chloroplast, fluorescence images from left to right. Scale bar = 100 μM.

Transiently expressed GmME1-YFP in Arabidopsis protoplast cells displayed fluorescence signal throughout the cytosol. The expressed YFP alone exhibited non-specific fluorescence within plasma membrane, cytosol and nucleus (**Figure 5**). Thus, GmME1 was suggested to localize at cytosol, which is similar to the cytosol localization of AtME2 and SgME1.

### Agrobacterium Mediated Over-expression of *GmME1* in Soybean Hairy Roots

**FIGURE 6:**
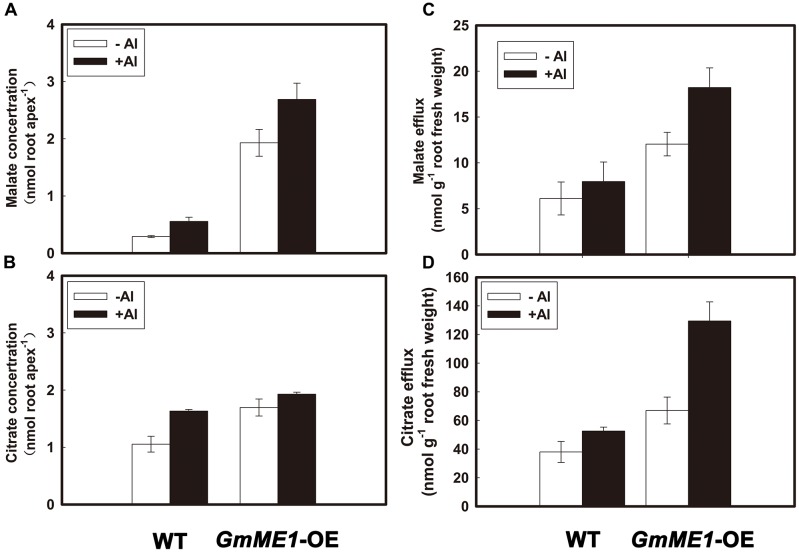
Concentration and efflux of malate **(A,C)** and citrate **(B,D)** in soybean hairy roots of *GmME1*-OE under Al stress. Both transgenic and WT hairy roots were exposed to 0.5 mM CaCl_2_ solution (pH 4.5) with or without 30 μM AlCl_3_ for 4 h. Ten root apices were excised from each treatment for malate **(A)** and citrate **(B)** concentration analysis. Root exudates were collected for malate **(C)** and citrate **(D)** efflux measurement. Fresh root biomass was weighed immediately. Data are given as the mean ± SD (*n* = 3).

In comparison with the WT, *GmME1*-OE hairy roots contained higher internal malate (**Figure 6A**) and citrate (**Figure 6B**) concentrations and secreted more malate (**Figure 6C**) and citrate (**Figure 6D**) under either -Al or +Al stress. Compared with that of WT under -Al treatment, nearly 10-fold higher malate concentrations were found in the *GmME1*-OE hairy roots (**Figure 6A**). Malate concentrations were further increased by Al treatment of both transgenic and WT hairy roots (**Figure 6A**). A slight but significant increase in citrate concentration was found in *GmME1*-OE hairy roots (**Figure 6B**). Different from WT hairy roots, the citrate concentration in *GmME1*-OE hairy roots could not be increased by Al treatment (**Figure 6B**). By sensitive enzymatic assay, malate and citrate were detected in the root exudates of both WT and *GmME1*-OE roots under either -Al or +Al treatment (**Figures 6C,D**). Compared with those of WT, 2.5-fold citrate efflux (**Figure 6D**) and 2.0-fold malate efflux (**Figure 6C**) increases were found in the root exudates of *GmME1*-OE hairy roots. The amount of malate efflux was approximately one-tenth that of citrate (**Figures 6C,D**).

**FIGURE 7:**
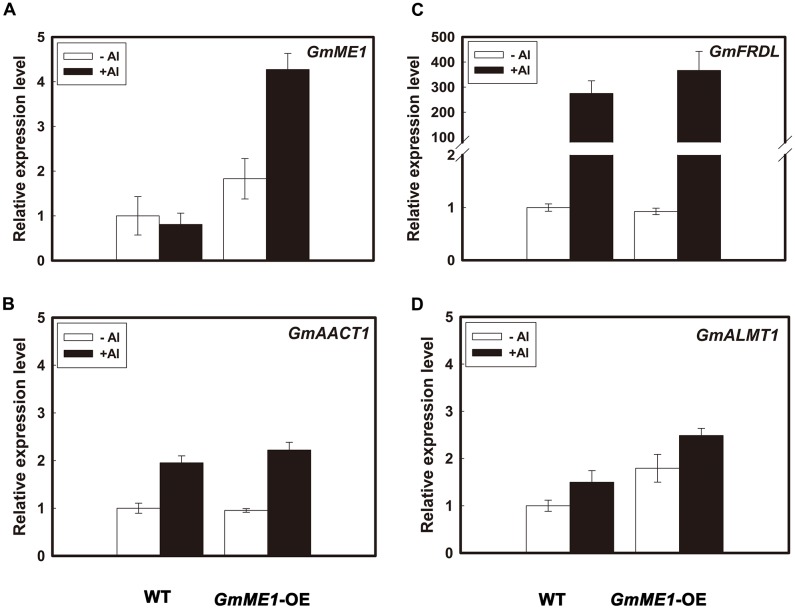
The transcriptional levels of *GmME1* **(A)**, *GmFRDL* **(B)**, *GmAACT1* **(C)**, and *GmALMT1* **(D)** in transgenic hairy roots under Al stress. Both transgenic and WT hairy roots were exposed to 0.5 mM CaCl_2_ solution (pH 4.5) with or without 30 μM AlCl_3_. Ten 0- to 1-cm root apices were excised from each treatment at 4 h for RNA isolation. Quantitative real time PCR was performed to study the transcriptional expression of **(A)** *GmME1* **(B)**, *GmFRDL*, **(C)** *GmAACT1* and **(D)** *GmALMT1*. Data are given as the mean ± SD (*n* = 3).

Transcriptional expression analysis showed that *GmME1*-OE hairy roots had higher transcriptional abundance of *GmME1* (**Figure 7A**). 4 h Al treatment didn’t cause significant changes of transcription abundance in *GmME1* in WT hairy roots, that was different from roots of Jiyu 62 response to Al stress (**Figure 3B**). The difference might result from distinct culture conditions or the different physiological properties between soybean roots and hairy roots. The over-expression of *GmME1* had less effect on the transcriptional patterns of either of the putative citrate transporters *GmAACT1* (**Figure 7B**) and *GmFRDL* (**Figure 7C**) or the malate transporter *GmALMT1* (**Figure 7D**).

**FIGURE 8:**
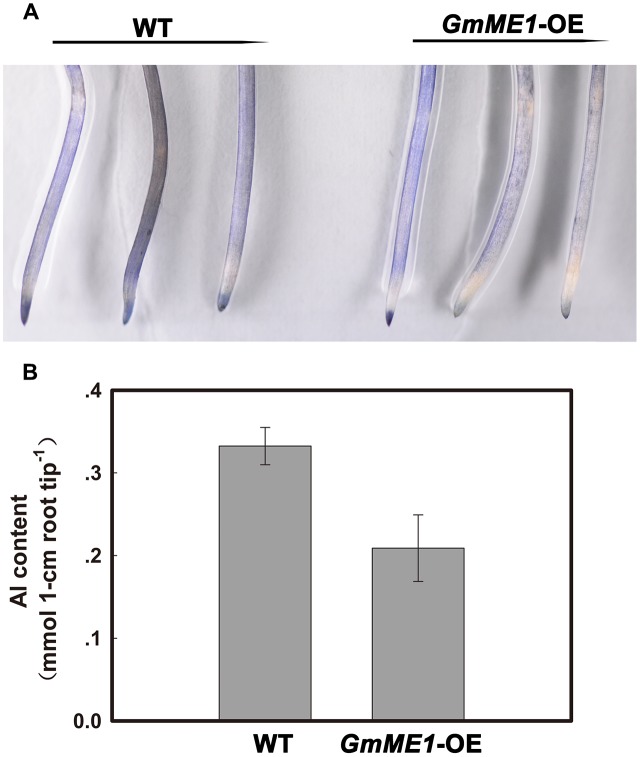
The hematoxylin staining **(A)** and Al content **(B)** in hairy roots under Al stress. Both transgenic and WT hairy roots were exposed to 0.5 mM CaCl_2_ solution (pH 4.5) with or without 30 μM AlCl_3_. At 4 h, three Al-treated WT or transgenic hairy roots were soaked in hematoxylin solution for 30 min. Roots were rinsed by water three times before imaging with a Nikon camera **(A)**. Ten 0- to 1-cm root apices were excised at 4 h for Al concentration measurements **(B)**.

Compared with the WT roots and in agreement with the higher organic acid concentration and exudation, lighter hematoxylin staining was found in the *GmME1*-OE roots after 4 h of Al treatment (**Figure 8A**). The Al content in the WT hairy roots was almost 1.5 fold that in the *GmME1*-OE hairy roots (**Figure 8B**). Thus, *GmME1*-OE hairy roots successfully acquired higher Al resistance.

## Discussion

Al-induced citrate exudation has been well documented as the Al–exclusion mechanism in soybean (Yang et al., 2000, 2001; Silva et al., 2001). In the present study, both genotypes showed sensitivity to Al with similarly lower citrate efflux before 8 h of Al treatment, and significantly more citrate efflux was induced from Jiyu 70 at 12 h, which resulted in its higher RRE (**Figures 1A,B**). Thus, the capacity for maintaining higher citrate exudation is critical for Al resistance in soybean. Malate was hypothesized to maintain the balance between the citrate synthesis and release in soybean root exposed to Al because the internal root concentration of citrate increased, whereas malate dropped (Yang et al., 2001). The sharp decrease in malate preceded citrate under Al stress in the Al-sensitive genotype Jiyu 62 (**Figures 2A,B**). The exhaustion of internal malate in Jiyu 62 was more consistent with its lower malic enzyme activities (**Figure 2C**) than the less Al-induced citrate efflux in Jiyu 62 (**Figures 1B**, **2B**). Thus, malate was suggested to play a crucial role in the sustained Al-induced citrate release from soybean roots. It is necessary to elucidate how malate metabolism affects the Al-induced citrate efflux from soybean roots.

Malate is one of the essential carbon storage molecules in plants (Zell et al., 2010) and has long been thought to be involved in regulating and composing the root exudates or affecting stomatal function as an osmolyte (Fernie and Martinoia, 2009). Malic enzyme reversibly converses between malate and pyruvate, depending on the isoform, cellular conditions and available substrates (Sweetman et al., 2009). Mitochondrial NAD-malic enzyme has been suggested to supply pyruvate for the TCA cycle to increase the citrate pool in common bean under Al stress (Rangel et al., 2010). Cytosolic isoforms of NADP-dependent malic enzymes have been found to regulate the cytosolic pH or stomatal closure by balancing malate synthesis and degradation (Martinoia and Rentsch, 1994; Laporte et al., 2002). Minimal changes in the primary structure of AtNADP-ME isoforms might result in very different kinetic behaviors of each AtNADP-ME isoform (Wheeler et al., 2008). Cytosol-localized NADP-ME2 and NADP-ME3 share 90% sequence identity but show distinct kinetic properties in their forward (malate oxidative decarboxylation) and reverse (pyruvate reductive carboxylation) reactions to regulation (Wheeler et al., 2009). SgME1 has been verified as a malic enzyme functioning in malate synthesis because of its over-expression in yeast, and *A. thaliana* and common bean hairy roots can significantly increase their malate concentrations (Sun et al., 2014). *GmME1* putatively encoding cytosolic NADP-dependent malic enzyme was revealed to increase its transcriptional abundance (You et al., 2011) and thus was chosen to study its contribution to Al-induced citrate efflux.

Fourteen homology genes putatively encoding cytosolic NADP-dependent malic enzyme existed in the soybean genome (**Figure 4B**). There have been no reports on their functional analysis until now. The transcription expression analysis in the present study revealed that *GmME1* was expressed throughout the whole soybean plant, at especially higher levels in the roots (**Figure 3A**). Al increased the transcription abundance of *GmME1* in soybean root apices of Jiyu 70 during 24 h (**Figure 3B**), which was consistent with its higher malate concentration (**Figure 2B**) and higher NADP-malic enzyme activities in the root apices (**Figure 2C**). GmME1 was localized to the cytosol (**Figure 5**) and displayed high similarity to AtME2 (77%), AtME3 (76%) and SgME1 (86%) (**Figure 4A**), which were conserved in the regions of suggested fumarate activation and malate inhibition (**Figure 4A**) (Wheeler et al., 2009). AtME3 is restricted to trichomes and pollen (Wheeler et al., 2009). According to sequence comparison (**Figures 4A,B**), subcellular localization (**Figure 5**), and spatial expression pattern (**Figure 3A**), GmME1 functions similarly to AtME2 and SgME1 as a malic enzyme contributing to malate metabolism.

Malate efflux from soybean under Al stress has been considered negligible because of several orders of lower magnitude and small variation between soybean genotypes (Yang et al., 2000; Silva et al., 2001). Soybean root malate exudation and concentrations were also reported to coordinately be influenced by pH changes, phosphorus deficiency, and Al toxicity (Liang et al., 2013). *GmALMT1* encoding a malate transporter was successfully cloned from soybean root apices, and GmALMT1-mediated root malate efflux was suggested to underlie soybean Al tolerance in soybean (Liang et al., 2013). The different conclusion in the role of Al-induced malate secretion might result from different experiment conditions and/or genotypes. Different from intact root treatment and HPLC detection in experiments from Yang et al. (2000, 2001), excised root apices and a sensitive enzyme assay were used in the experiments of Liang et al. (2013). In the present study, malate efflux was not detected by HPLC from Al-induced root exudates in 14-day-old seedlings of Jiyu 62 and Jiyu 70 (data not shown). However, malate exudation could be detected in the hairy root experiment by the enzyme assay (**Figures 6C,D**), although it was approximately one-tenth that of citrate efflux.

Over-expressing *GmME1* in soybean hairy roots enhanced its own expression (**Figure 7A**) and resulted in a significant increase in malate and citrate concentrations under either +Al or -Al treatment (**Figures 6A,B**). Al-induced malate efflux was found to increase in the *GmME1*-OE hairy roots, although the transcription level of *GmALMT1* encoding malate transporter remained constant (**Figure 7D**). Thus, GmME1 was verified to be a malic enzyme similar to SgME1, responsible for malate synthesis and efflux under Al stress (Sun et al., 2014).

Citrate efflux was approximately 10-fold higher than malate efflux in the hairy roots of transgenic and wild-type plants (**Figures 6C,D**). With unchanged transcription levels of *GmFRDL* and *GmAACT1* putatively encoding citrate transporter (**Figures 7B,C**), more Al-induced citrate secretion was found from *GmME1*-OE root exudates than that of WT (**Figure 6D**), which might have resulted from their more internal citrate concentration (**Figure 6B**). GmME1, a malic enzyme, increased the citrate synthesis and citrate efflux, which supports our previous hypothesis that malate contributes to balance citrate synthesis and efflux in soybean. Discussion of how GmME1 affects the citrate pool and then efflux follows.

**FIGURE 9:**
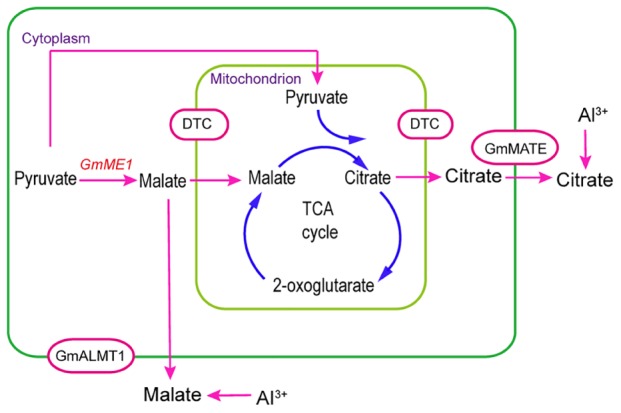
A Model proposing the role of *GmME1* in the process of Al-induced malate and citrate efflux from soybean roots. Cytosolic malic enzyme, GmME1, was supposed to increase malate synthesis in cytosol and thus supply malate to the TCA cycle as an anaplerotic reaction to promote citrate pool over citrate exudation under Al stress. Imported malate and exported citrate through mitochondrial membrane were supposed to occur by GmDTC. The malate and citrate efflux across plasma membrane might have occurred by GmALMT1 and MATE family proteins (GmFRDL and GmAACT1), respectively.

Mitochondrial TCA cycle-related enzymes, especially citrate synthase, have been proposed to prompt the Al-induced citrate efflux from soybean roots (Xu et al., 2010). Anaplerotic enzyme induction has been revealed to counteract the depletion of TCA intermediates. Root isoforms of PEPC and NAD malic enzyme are known to have various anaplerotic functions involved in carbon skeleton supply during N assimilation, maintenance of cytoplasmic pH or osmolarity regulation (Nisi and Zocchi, 2000; Held, 2005). Both PEPC and mitochondrial NAD-ME were proposed to be involved in anaplerotic functions in common bean under Al stress by fueling the TCA cycle (Rangel et al., 2010). This means that the anaplerotic reaction is necessary for some plant species under Al stress. One report on hypertrophied hearts suggested that cytosolic malic enzyme catalyzes pyruvate carboxylation to supply more malate to the mitochondrial TCA cycle, leading to more citrate synthesis (Pound et al., 2009). “Anaplerotic” influx depends on the direct shuttle of malate between the cytosol and mitochondria (Pound et al., 2009). In the present study, *GmME1*-OE hairy roots contained more malate, which prompted more synthesis and secretion of citrate (**Figures 6B,C**). This process is depicted in **Figure 9**. Cytosol-localized GmME1 might be involved in an alternate anaplerotic pathway to supply the TCA under Al stress by promoting more malate synthesis (**Figure 9**). Mitochondrial carrier proteins function to export or import metabolite to maintain the pools of TCA cycle intermediates (Haferkamp and Schmitz-Esser, 2012; Etienne et al., 2013). Dicarboxylate/tricarboxylate carrier (DTC) was suggested to transport dicarboxylates, such as oxaloacetate and malate, and tricarboxylates, including citrate, isocitrate, *cis*-aconitate, and trans-aconitate across mitochondrial membrane by a counter-exchange mechanism (Deng et al., 2009). CjDTC was suggested to involve in organic acid excretion in *Citrus junos* because of its higher expression under Al stress (Deng et al., 2009). Consistently, the expression of *DTC* was increased in soybean root apices under Al stress (You et al., 2011). Citrate carrier inhibitor treatment decreased Al-induced citrate efflux from soybean, indicating its important role in the process of citrate release (Xu et al., 2010). In this paper, the import of malate and export of citrate in mitochondria might depend on mitochondrial carrier proteins such as DTC protein (**Figure 9**). The malate and citrate efflux across the plasma membrane was supposed to depend on GmALMT1 and members of the MATE family (GmAACT and GmFRDL) (**Figure 9**).

Consistent with the increased efflux and concentration of both malate and citrate, the *GmME1*-OE soybean hairy roots have light hematoxylin staining (**Figure 8A**) and lower Al contents (**Figure 8B**) in root apices, demonstrating better Al exclusion capacity and higher Al resistance.

## Conclusion

*GmME1* was revealed to encode a cytosolic malic enzyme, which increased malate and citrate synthesis and Al-induced malate and citrate efflux. Moreover, new evidence was added that GmME1 can function in anaplerotic pathways to supply the TCA cycle to prompt more citrate synthesis then efflux under Al stress (**Figure 9**).

## Author Contributions

YZ performed most of the experiments. ZY the supervisor of YZ, helped to design the experiments and performed organic acid measurement by HPLC. YX performed the subcellular localization experiments. BL and WS helped in plant culture and hairy root induction experiments. HS and ZS helped in qRT-PCR experiments. JY designed the entire experiment, performed organic acid examination by enzymatic assay, and wrote the manuscript. The final manuscript has been read and approved by all authors.

## Conflict of Interest Statement

The authors declare that the research was conducted in the absence of any commercial or financial relationships that could be construed as a potential conflict of interest.

## Footnotes

1 http://primer3.ut.ee/
